# Abstracts

**DOI:** 10.1155/S1463924683000176

**Published:** 1983


### Abstracts

### Page 68

### Introduction to cost-benefit analysis
R. Haeckel

Economic aspects of the decision-making process for the purchase of instrumentation are outlined with respect to cost-benefit and cost-effectiveness analysis. Simplified procedures for cost comparison are described.

### Introduction à l'analyse frais/bénéfice

R. Haeckel

Les aspects économiques du processus de prise de décision pour l'achat d'instruments sont décrits par rapport aux frais/bénéfice et efficacité. Des procédures simplifiées pour la comparaison des frais sont décrites.

### Einführung in die Kosten-Nutzen-Analyse
R. Haeckel

Ökonomische Aspekte des Entscheidungsprozesses bei der Anschaffung von Analysengerätenwerden in Bezug auf Kosten-Nutzen- und Kosten-Wirksamkeits-Analysen dargelegt. Vereinfachte Verfahren des Kostenvergleiches werden beschrieben.

---

### Page 71

### Special problems of cost analysis and cost-benefit analysis as applied to large multi-test analysers
J. E. Barclay

Industrial management techniques such as cost analysis and cost-benefit analysis may be applied to the clinical laboratory. Large multi-test analysers have a major impact on laboratory organization. Cost analysis and cost-benefit analysis of such instruments must take into account all the organizational changes; including staff structure and number, and ancillary equipment required around the analyser. The impact of instrument features on the allocation of costs is discussed and a suggestion made on the method of comparison of alternative instruments and organizations.

### Problèmes particuliers lies à l'analyse des coûts et à l'analyse de coût et rendement appliques aux grands analyseurs multiparametriques

J. E. Barclay

Les techniques industrielles d'administration telles que l'analyse des coûts et l'analyse de coût rendement peuvent s'appliquer au laboratoire d'analyses médicales. Un analyser multiparamétrique modifie considérablement l'organisation du laboratoire. Dans l'analyse des coûts et des avantages économiques de tels systèmes, on doit prendre en compte tous les changements d'organisation, notamment en ce qui concerne le personnel (effectif et structure de l'encadrement) et l'équipement auxiliaire nécessaire à l'utilisation de l'analyseur.

L'impact des caractéristiques du système sur la répartition des coûts est présenté. Des suggestions ont été faites concernant la comparaison avec d'autres systèmes et d'autres organisations.

### Spezielle Probleme der Kosten- und Kosten/Nutzen-Analyse von grossen Multitest-Analysatoren
J. E. Barclay

Industrielle Management-Verfahren wie Kosten- und Kosten/Nutzen-Analysen können auch im klinischen Laboratorium angewandt werden. Grosse Multitest-Analysatoren haben einen grossen Einfluss auf die Organisation eines Laboratoriums. Kosten- und Kosten/Nutzen-Analysen solcher Geräte müssen auch organisationelle Änderungen berücksichtigen, wie Organigramm und Zahl der Mitarbeiter, sowie Hilfsgeräte, die in diesem Zusammenhang benötigt werden.

Der Einfluss der Geräteeigenschaften auf die Kostenzuordnung wird dargestellt und es wird ein Vorschlag gemacht, wie verschiedene Geräte und Organisationen miteinander verglichen werden können.

---

### Page 75

### Establishing and maintaining the data-base required for cost analysis in a clinical laboratory
G. E. Westlake

Before costing can be accurately carried out in any clinical laboratory the individual costs must be available from the accounting system. Whilst many hospitals have costing systems for many reasons, these do not fit the bill. The paper discusses these inadequacies in the current systems and then details the requirements for a correct accounting and costing system. The author's wide experience of data-bases is used in this process.

### Establissement et soutien de la base de données nécessaires pour l'analyse de frais dans un laboratoire clinique
G. E. Westlake

Avant qu'une adéquate calculation peut être exécuté dans n'importe quel laboratoire clinique, les frais individuels doivent être présentés par la comptabilité. Beaucoup d'hôpitaux ont des systèmes de comptabilité qui, pour différentes raisons, ne sont pas applicables. L'article discute les désaccords dans les systèmes actuels et traîte en détail les éxigences à un système de comptabilité et de frais correct. La vaste expérience de l'auteur dans le domaine des données est utulisée dans ce processus.

### Einrichtung und Unterhalt einer Datenbank für die Kostenanalyse in einem klinischen Labor
G. E. Westlake

Bevor in einem klinischen Labor eine genaue Kostenverrechnung durchgeführt werden kann, müssen vom Rechnungswesen die individuellen Kosten erfasst werden. Während viele Spitäler ein Kostenverrechnungssystem für die verschiedensten Zwecke unterhalten, ist dieser Punkt im Menü nicht enthalten. Die vorliegende Arbeit bespricht die Unzulänglichkeiten der bestehenden Systeme und detailliert nachfolgend die Anforderungen an ein korrektes Kostenerfassungs- und Verrechnungssystem. Die grosse Erfahrung des Autors mit Datenbanken wird dabei extensiv genutzt.

---

### Page 79

### Cost accounting in the EDP-supported chemical laboratory
H. J. Gibitz

The chemical central laboratory at the Salzburg Landeskrankenstalten, a 1600-bed hospital, is divided into 10 cost centres: eight of these are functional laboratory sections and two are general sections. All primary and secondary costs were calculated exactly for every cost centre and then compared to the number of tests performed in one year. The average cost of a result can differ substantially: a test in the autoanalyser laboratory collects 15 cost points; in the mechanized enzyme-laboratory: 19; in the manual laboratory (simple tests): 54; in the protein laboratory: 70; in the emergency laboratory: 68; in endocrinology: 88; in the manual laboratory (special tests): 216; and in clinical toxicology: 793 cost points.

The cost account of the whole hospital shows that the chemical central laboratory represented 2·91% of total cost in 1979. The costs for personnel for the whole hospital amounted to 58% of the total and those for the central laboratory were 38%.

Three years' experience shows an average annual increase of costs of 10·3% for all tests in the central laboratory. In two of the eight laboratory cost centres the costs per test reduced. In the autoanalyser laboratory a decrease of 24% is due to on-line data processing of the analogue data from the autoanalysers which are connected to a new IBM System 7. An exact analysis of the variable and non-variable costs for all the tests performed in the autoanalyser laboratory shows that applying equal costs to all the tests at the cost centre does not result in any significant error.

In the toxicology laboratory a 35% decrease in the cost per test is caused by an increase in the number of single enzyme immunoassays, which are cheaper than the toxicological drug screenings carried out by thin layer chromatography.

The costs of all tests executed in the central laboratory are allocated to the clinical department which requested the test. The costs per clinical department, per test and bed and per single test differ widely. This is due to the different sizes of and the various medical tasks performed in the clinical departments.

Cost analysis is an important tool for laboratory management; its results are important to future investigations about the non-monetary utility of laboratory tests.

### La comptabilité dans le laboratoire chimique soutenue par l'informatique
H. J. Gibitz

Le laboratoire chimique central de notre hôpital de 1600 lits est divisé en 10 centres de frais: 8 centres correspondent à des unités de laboratoire fonctionnelles, 2 sont de genre général. Tous les frais directs et indirects sont calculés exactement pour chaque section est sont confrontés avec la quantité de tests exécutés. Les frais moyens par test diffèrent considérablement: laboratoire autoanaliseur 15 unités de frais, laboratoire enzymes méchanisé 19, laboratoire manuel pour tests simples 54, laboratoire de protéines 70, laboratoire cas urgents 61, laboratoire endocrineologique 102, laboratoire manuel pour tests spéciaux 216, toxicologie clinique 793 unités de frais.

L'évaluation des frais de l'hôpital entier prouve que la quote-part des frais du laboratoire central s'est monté en 1979 à 2,91%. Dans l'hôpital en général les frais du personnel se montaient à 58%, au laboratoire central à 38%.

La comparaison des frais pendant 3 ans montre pour le laboratoire central une augmentation de 10,3% par test. Dans 2 de 8 sections les frais par test ont diminués. La diminution de 24% au laboratoire autoanaliseur est la suite de la connection on-line des instruments d'analyse au processeur IBM S7. Une analyse exacte des frais variables et non-variables pour chaque analyse du laboratoire autoanaliseur montre que l'erreur est minime si pour chaque analyse d'un secteur les mêmes frais sont utilisés.

La diminution des frais totaux de 35% au laboratoire de toxicologie est due à l'augmentation extrèmement forte des 'enzyme immuno assay' dont les frais par test sont plus bas que les frais pour le 'toxicological drug screening' au moyen de la chromatographie à couche fine.

Les frais de toutes les analyses exécutées au laboratoire central sont transmis aux stations de malades qui ont demandé les analyses. Les frais totaux par station de malades, les frais par test et lit de malade et les frais par test individuel montrent de claires différences qui ne sont pas seulement dues à la taille différente et les nécessités médicales diverses des stations de malades.

L'évaluation des frais est un instrument important pour le management du laboratoire. Ses résultats forment une condition nécessaire pour des recherches futures sur le profit nonmonétaire d'analyses de laboratoire.

### Kostenverrechnung in einem EDV-unterstützten Chemielabor
H. J. Gibitz

Das chemische Zentrallaboratorium der Landeskrankenanstalten Salzburg (1600-Betten) ist in 10 Kostenstellen gegliedert, 8 Kostenstellen entsprechen funktionellen Laboreinheiten, 2 sind allgemeiner Art. Alle direkten und indirekten Kosten werden für jede Kostenstelle exakt berechnet und der Anzahl der durchgeführten Tests gegenübergestellt. Die mittleren Kosten pro Test unterscheiden sich beträchtlich: Autoanalyzer-Laboratorium: 15, mechanisertes Enzymlaboratorium 19, manuelles Laboratorium für einfache Tests: 54, Proteinlaboratorium: 70, Notfallaboratorium: 61, endokrinologisches Laboratorium: 102, manuelles Laboratorium für Spezialtests: 216, klinische Toxikologie: 793 Kosteneinheiten.

Die Kostenrechnung des gesamten Krankenhauses ergibt, daß der Anteil der Kosten des Zentrallaboratoriums im Jahre 1979 2,91% betrug. Im gesamten Krankenhaus entfielen auf Personalkosten 58%, im Zentrallaboratorium 38%.

Der Kostenvergleich während 3 Jahren zeigt für das Zentrallaboratorium eine Zunahme von 10,3% pro Test. In 2 von 8 Kostenstellen haben die Kosten pro Test abgenommen. Die Abnahme von 24% ist im Autoanalyzer-Laboratorium eine Folge des on-line Anschlusses der Analysengeräte an den Prozessrechner IBM S7. Eine exakte Analyse der variablen und nicht variablen Kosten für jede Untersuchung des Autoanalyzer-Laboratoriums zeigt, daß sich der Fehler in vertretbaren Grenzen hält, wenn für alle Untersuchungen einer Kostenstelle die gleichen Kosten benützt werden.

Die Abnahme der Gesamtkosten im toxikologischen Laboratorium um 35% beruht auf der besonders starken Zunahme von Enzymimmunoassays, deren Kosten pro Test niedriger sind, als die Kosten für das Toxicological Drug Screening mittels Dünnschichtchromatographie.

Die Kosten aller im Zentrallaboratorium durchgeführten Untersuchungen werden an die Krankenabteilungen weitergegeben, die die Untersuchungen beantragt haben. Die Gesamtkosten pro Krankenabteilung, die Kosten pro Test und Krankenbett und die Kosten pro Einzeltest zeigen deutliche Unterschiede, die nicht nur durch die unterschiedliche Größe und die verschiedenen medizinischen Aufgaben der Krankenabteilungen bedingt sind.

Die Kostenrechnung ist ein wichtiges Instrument für das Laboratoriumsmanagement. Ihre Ergebnisse bilden auch eine notwendige Voraussetzung für zukünftige Forschungen über den nicht monetären Nutzen von Laboratoriumsuntersuchungen.

### Page 83

### A microcomputer-based high-speed data-acquisition system for a stopped-flow spectrophotometer: PDP-11 architecture interfaced to a commercial instrument

J. A. Alcocer *et al.*

A microcomputer-based data-acquisition system is described for use with a stopped-flow spectrophotometer such as the commercially available Durrum-Gibson and others of similar design. The microcomputer constructed was based on the Digital Equipment Corporation's LSI-11/2 microprocessor, which uses architecture of the PDP-11 series. An easily implemented interface is described for high-speed 12-bit analogue-to-digital conversions of absorbance data from the stopped-flow spectrophotometer. The system was evaluated using an iron (III)-thiocyanate reaction to yield a first-order rate constant of 15·0/s. The reaction of oxymyoglobin with sodium dithionite was also studied on this system and gave a first-order oxygen-dissociation constant of 23·0/s with a calculated half-time of 30·2 ms.

A data-handling routine which plots data on a graphics display terminal is described. The software is interactive and allows the data to be evaluated easily and quickly.

### Une système d'acquisition de données pour un spectrophotomètre à flux interrompu basé sur un microordinateur à haute vitesse: architecture PDP-11 interfacé à un instrument commercial

J. A. Alcocer *et al.*

Un système d'acquisition de données basé sur microordinateur est décrit pour l'utilisation avec un spectrophotomètre 'stopped-flow' tel que le Durrum-Gibson et autres instruments commerciaux de construction similaire. Le microordinateur était basé sur le microprocesseur LSI-11/2 de la Digital Equipment Corporation qui utilise l'architecture de la série PDP-11. Une interface simple pour la conversion 12-bit analogue/digital à haute vitesse des données d'absorption du spectromètre 'stopped-flow'. Le système a été évalué en utilisant la réaction de fer (III) thiocyanate qui donne une constante de vitesse de première ordre de 15·0/s. La réaction du oxymyoglobine avec la dithionite de sodium a également été étudiée sur ce système et donne une constante de dissociation pour l'oxygène de première ordre de 23·0/s avec un mi-temps calculé de 30·2 ms. Une routine pour traitement de données qui présente les données sur un terminal avec écran graphique est décrite. Le software est interactif et permet d'évaluer les données facilement et rapidement.

### Ein schnelles Mikroprozessor-Datenerfassungssystem für ein stopped-flow Spektrophotometer: PDP-11 Architektur gekoppelt mit einem kommerziellen Instrument

J. A. Alcocer *et al.*

Es wird ein schnelles Mikroprozessor-Datenerfassungssystem beschrieben, das mit einem stopped-flow Spektrophotometer wie das kommerziell erhältliche Durrum-Gibson und anderen ähnlicher Konstruktion verwendet werden kann. Der konstruierte Mikrocomputer basiert auf dem LSI-11/2 Mikroprozessor der Digital Equipment Corporation, der die Architektur der PDP-11 Serie verwendet. Ferner wird ein leicht zu bauendes Interface beschrieben, das die schnelle 12-bit analog/digital Konversion der Absorptionsdaten des stopped-flow Spektrophotometers erleichtert. Das System wurde mit der Eisen (III)-Thiocyanat Reaktion getestet, die eine Reaktionskonstante erster Ordnung von 15,0/s ergab. Daneben wurde mit diesem System die Reaktion von Oximyoglobin mit Natriumdithionit untersucht. Dabei wurde für die Reaktionskonstante erster Ordnung der Sauerstoff-Dissoziation 23,0/s erhalten und eine halbwärts Zeit von 30,2 ms errechnet.

Die Beschreibung eines Datenverarbeitungsprogramms zeigt, wie Daten auf einem graphischen Bildschirm aufgezeichnet werden können. Die Programmierung ist interaktiv gestaltet und erlaubt eine leichte und schnelle Auswertung der Daten.

### Page 89

### High-performance liquid chromatographic analysis of water-soluble vitamins in tablets with automatic continuous-flow sample preparation

S. C. Coverly

The analysis of nicotinamide, pyridoxine (vitamin B6), riboflavin (vitamin B2) and thiamin (vitamin B1) in solid pharmaceutical products is described using the Technicon 'FAST-LC' system. Tablets are dissolved in an aqueous buffer and solids are separated from the vitamin solution by dialysis before injection onto the chromatographic column. Chromatograms are monitored at 272 nm at analysis rates of five to 10/h with average CVs of 2 to 4%. The system was tested with nine pharmaceutical products of different composition. Precision and recovery data for one product are presented and other applications of the system are referred to.

### Chromatographie liquide à haute performance des vitamines solubles dans l'eau dans des dragées avec préparation d'échantillon à flux continu automatique

S. C. Coverly

L'analyse de nicotinamide, pyridoxine (vitamine B6), riboflavine (vitamine B2) et thiamine (vitamine B1) dans des produits pharmaceutiques solides est décrite utilisant le système Technicon FAST-LC. Les dragées sont dissolues dans un tampon aqueux et les particules solides sont séparées de la solution de vitamines par dialyse avant l'injection sur la colonne chromatographique. Les chromatogrammes sont détectées à 272 nm et le nombre d'analyses était de 5 à 10 par heure avec un coéfficient de variation moyen de 2 à 4%. Le système a été testé avec 9 produits pharmaceutiques de différente composition. La précision et le pourcentage retrouvé pour un produit sont présentés et d'autres applications du système sont mentionnées.

### Hochleistungs-Flüssigchromatographieanalyse mit kontinuierlicher Probenvorbereitung von wasserlöslichen Vitaminen in Tabletten

S. C. Coverly

Die Arbeit beschreibt die Analyse von Nikotinamid, Pyridoxin (Vitamin B6), Riboflavin (Vitamin B2) und Thiamin (Vitamin B1) in festen pharmazeutischen Produkten unter Verwendung des Technicon FAST-LC Systems. Tabletten werden in einem wässrigen Buffer gelöst und Feststoffe werden von der Vitaminlösung vor der Injektion in die Chromatographie-Kolonne durch Dialyse abgetrennt. Die Chromatogramme werden bei einem Durchsatz von 5–10 pro Stunde und einem durchschnittlichem CV von 2–4% bei 272 nm detektiert. Das System wurde mit 9 pharmazeutischen Produkten verschiedener Zusammensetzung getestet. Für ein Produkt werden Präzision und Daten dargestellt, und auf andere Applikationen des Systems wird hingewiesen.

### Page 94

### A versatile automatic gas-sampling system
H. L. Reed *et al.*

An automatic gas-sampling system has been designed in which a number of evacuated test-tubes are sequentially exposed to the atmosphere at regular predetermined intervals. The operating sequence of the simple, low-cost mechanism is controlled by a 6802 microprocessor. This portable unit has been designed to determine air infiltration into chillers, freezers and cold stores by measurement of the concentration decay of a tracer gas during a series of time intervals. Although specifically designed for gas sampling, minor modifications to the mechanism would enable the unit to be used for the sampling of liquids.

### Un système automatique et versatile pour l'échantillonnage de gaz

H. L. Reed *et al.*

Un système automatique pour échantillonnage de gaz a été construit dans lequel un nombre de tubes évacués sont exposés à l'atmosphère avec des intervalles réguliers prédéterminés. Une séquence d'opération de ce mécanisme simple et bon marché est contrôlée par un microprocesseur 6802. Cette unité portable a été construite pour déterminer l'infiltration d'air dans des systèmes de refroidissement et de congélation en mesurant la diminution de la concentration d'un gaz de traçage pendant une série d'intervalles de temps. Bien que construit spécialement pour l'échantillonnage de gaz, de petites modifications du mécanisme permettraient d'utiliser cette unité pour l'échantillonnage de liquides.

### Ein vielseitiges automatisches Probenahme-System für Gase
H. L. Reed *et al.*

Es wird die Konstruktion eines vielseitigen automatischen Probenahme-Systems für Gase beschrieben, bei welchem eine Reihe von evakuierten Reagenzgläsern sequentiell in regelmässigen und vorgängig festgesetzten Intervalen der Atmosphäre ausgesetzt werden. Der Betriebsablauf des einfachen und billigen Mechanismus wird durch einen 6802 Mikroprozessor gesteuert. Diese tragbare Einheit wurde so konstruiert, dass damit die Luftinfiltration von Kühleinheiten, Gefrieranlagen und Kaltlagerräumen mit Hilfe der Bestimmung des Konzentrationsabfalls eines Spurengases während einer Reihe von Zeitintervalen bestimmt werden kann. Obwohl speziell für die Probenahme von Gasen konstruiert, würde die Einheit mit kleineren Änderungen am Mechanismus auch das Probenziehen von Flüssigkeiten erlauben.

### Page 99

### Automated chemical synthesis. Part 2: Interfacing strategies
D. F. Chodosh *et al.*

New highly automated tools for the chemical laboratory will enhance research productivity and extend research capabilities. In order to more quickly and comprehensively characterize and evaluate synthetic routes an automated bench-scale 'batch-type' chemical reactor capable of self-directing experimentation and optimization has been designed. After evaluating the use of a remote time-share computer environment a dedicated real-time minicomputer was selected and interfaced to this synthesis system to provide instrument control, on-line optimization calculations and data-acquisition capabilities. The authors explore and contrast the time-share and dedicated computing environments and detail the interfacing problems associated with implementing each environment. To avoid catastrophe in the laboratory a fail-safe interface has been designed, which couples the synthesis apparatus with the computing system.

### Synthèse chimique automatique. II: Stratégies d'interfaçage

D. F. Chodosh *et al.*

De nouveaux instruments hautement automatisés pour le laboratoire chimique vont augmenter la productivité en recherche et permettre de nouveaux domaines de recherche. Pour permettre de mieux caractériser et évaluer des nouveaux chemins synthétiques, nous avons développé un réacteur chimique automatique pour laboratoires capable de se contrôler soi-même et optimaliser des expériences. Après évaluation d'un ordinateur éloigné en mode 'time-share' nous avons choisi et interfacé un mini-ordinateur en temps réel dédié au système de synthèse pour permettre le contrôle de l'instrument, le calcul d'optimisation en ligne et l'acquisition des données. Dans cet article nous étudions et comparons le système d'ordinateur 'time-share' et dédié et discutons en détail les problèmes d'interface associés avec les deux systèmes. Pour éviter une catastrophe dans le laboratoire, nous avons construit une interface avec très grande sécurité qui couple l'appareil de synthèse avec le système d'ordinateur.

### Automatisierte chemische Synthese. II: Interface-Strategien
D. F. Chodosh *et al.*

Neue hochautomatisierte Werkzeuge für das chemische Laboratorium werden die Produktivität der Forschung fördern und die Möglichkeiten der Forschung ausweiten. Um schneller und vollständiger synthetische Wege zu charakterisieren und zu beurteilen, haben wir einen automatisierten, diskontinuierlichen chemischen Reaktor für den Labortisch entworfen, der der selbstgesteuerten Experimentierung und Optimierung fähig ist. Nach Evaluation der Benützung eines 'time-share'-Computersystems entschieden wir uns für einen zweckgebundenen Minicomputer, der mit Interfacegeräten an dieses System für die Steuerung, on-line Optimierungsberechnungen und Datenerfassung angeschlossen wurde. In dieser Arbeit betrachten und vergleichen wir das Vorgehen mit 'time-sharing' System und zweckgebundenem Computer, und wir detaillieren die Interfaceprobleme, die mit den beiden Lösungen verbunden sind. Um Katastrophen im Labor zu vermeiden, haben wir ein ausfallsicheres Interface entworfen, dass die Syntheseapparatur mit dem Rechnersystem verbindet.

### Page 103

### Automated chemical synthesis. Part III: Temperature control systems

F. Chodosh *et al.*

A computer-controlled 'batch-type' reactor system has been designed which will permit rapid evaluation of chemical reactions. The accuracy, precision, safety and efficiency of the temperature control of the system are of fundamental concern if meaningful results are to be obtained via automated experimentation. Interfaces have been constructed that permit the dedicated computer system to control heating and cooling systems which, acting in concert, afford reliable temperature control of the mini-vessel. Interfacing strategies and concepts are described.

### Synthèse chimique automatique. III: Système de contrôle de la température

D. F. Chodosh *et al.*

Un système de réaction discontinu contrôlé par ordinateur a été construit qui permet l'évaluation rapide de réactions chimiques. L'exactitude, la précision, la sûreté et l'efficacité du contrôle de température du système sont d'importance fondamentale pour obtenir des résultats significatifs pour expériences automatiques. Des interfaces ont été construites qui permettent au système d'ordinateur de contrôler le système de chauffage et de refroidissement qui, actionnés ensemble, permettent un contrôle de température du minirécipient sans faute. La conception et la stratégie de l'interface sont décrites.

### Automatisierte chemische Synthese. III: Temperaturregulierung

D. F. Chodosh *et al.*

Es wird ein computergesteuertes, diskontinuierliches Reaktorsystem beschrieben, welches die rasche Beurteilung von chemischen Reaktionen erlaubt. Die Genauigkeit, Präzision, Sicherheit und Effizienz der Temperaturregulierung des Systems sind von fundamentaler Bedeutung, wenn mittels automatisierter Experimentierung nützliche Resultate erhalten werden sollen. Die gebauten Interface-Geräte erlauben dem Computersystem die Steuerung der Heiz- und Kühlsysteme, welche aufeinander abgestimmt, eine zuverlässige Temperaturregulierung des Minikessels möglich machen. Interface-Strategien und Konzepte sind beschrieben.

### Page 108

### Computer interface for a diode-array based spectrophotometer

H. L. Pardue *et al.*

This paper describes hardware and software developed to interface the Hewlett-Packard 8450A spectrophotometer to a general-purpose computer. Selected data for the reaction of acetoacetate with glycine are presented to illustrate the utilization of rapid scanning, multiwavelength kinetic data obtained with the system.

### Interface d'ordinateur pour un spectrophotomètre basé sur un 'diode array'

H. L. Pardue *et al.*

Cette publication décrit l'instrumentation et les programmes développés pour interfacer un spectrophotomètre Hewlett-Packard 8450A avec un ordinateur d'utilité générale. Des données choisies pour la réaction d'acétoacétate avec la glycine sont présentées pour illustrer l'utilisation de données cynétiques obtenues avec ce système avec changement rapide de la longueur d'onde.

### Rechner-Interface für ein Dioden-Array Spektrophotometer

H. L. Pardue *et al.*

Diese Arbeit beschreibt die Hardware und Software, die entwickelt wurde, um ein Hewlett-Packard 8450A Spektrophotometer an einen Allzweckrechner zu koppeln. Anhand von Daten für die Reaktion von Acetoacetat mit Glycin wird die Verwendung der raschen Aufnahme kinetischer Daten bei gleichzeitig vielen Wellenlängen, die mit diesem System möglich ist, illustriert.

### SEMINAR ANNOUNCEMENT

*Practical sampling systems for on-line process measurement*

Organized by Sira, and to be held on 18 May 1983 at the Meeting Rooms of the Zoological Society of London, Regent's Park, London NW1, the seminar is a follow-up to those held in 1981 and 1978 on the same topic. The objective is to provide an overall view of the use of sampling systems for on-line process measurement for liquids and gases, and also for slurries, pastes and solids, and to show through practical examples the methods which have been adopted in various industries.

The morning session will consist of four keynote lectures covering the use of sampling systems for liquids and gases; sampling of flue gases, sampling of slurries, pastes and solids; and sampling for environmental monitoring. The afternoon session will include presentations by speakers from a range of industries, and a group discussion period in which speakers will lead groups in the discussion of a variety of topics of interest to delegates.

The seminar is primarily intended for instrument engineers, plant laboratory analysts and process control engineers.

Literature about commercially available equipment will be displayed at the seminar.

*Details from the Conference Unit, Sira Institute Ltd, South Hill, Chislehurst, Kent BR7 5EH, UK. Tel.: 01 467 2636.*

